# From mitochondrial DNA arrangement to repair: a kinetoplast-associated protein with different roles in two trypanosomatid species

**DOI:** 10.1186/s13071-025-06985-8

**Published:** 2025-08-28

**Authors:** Camila Silva Gonçalves, Carolina Moura Costa Catta-Preta, Bruno Marçal Repolês, Wesley Roger Rodrigues Ferreira, Flavia Souza Morini, Jeremy C. Mottram, Danielle Pereira Cavalcanti, Wanderley de Souza, Stenio Perdigão Fragoso, Carlos Renato Machado, Maria Cristina M. Motta

**Affiliations:** 1 Laboratório de Ultraestrutura Celular Hertha Meyer, Centro de Pesquisa em Medicina de Precisão (CPMP), Instituto de Biofísica Carlos Chagas Filho, Universidade Federal Do Rio de Janeiro, Cidade Universitária, Rio de Janeiro, RJ CEP 21941-590 Brazil; 2 Centro Nacional de Biologia Estrutural e Bioimagem – CENABIO, Rio de Janeiro, RJ Brazil; 3 Department of Biology, York Biomedical Research Institute, University of York, Wentworth Way, Heslington, York, YO10 5DD UK; 4 Departamento de Bioquímica e Imunologia, Laboratório de Genética Bioquímica, Instituto de Ciências Biológicas, Universidade Federal de Minas Gerais, Belo Horizonte, Brazil; 5 Laboratório de Biologia Molecular e Sistêmica de Tripanossomatídeos, Instituto Carlos Chagas, Fundação Oswaldo Cruz (FIOCRUZ), Curitiba, Brazil; 6 Laboratório de Microbiologia, Diretoria de Metrologia Científica, Industrial e Tecnologia, Instituto Nacional de Metrologia, Qualidade e Tecnologia - Inmetro, Duque de Caxias, RJ Brazil

**Keywords:** Trypanosomatids, kDNA replication and topology, Genotoxic agents, Kinetoplast-associated proteins (KAPs), Symbiosis, Cell proliferation, Ultrastructure

## Abstract

**Background:**

One of the most intriguing and unusual features of trypanosomatids is their mitochondrial DNA, known as kinetoplast DNA (kDNA), which is organized into a network of concatenated circles. The kDNA is contained within the mitochondrial matrix and can exhibit distinct arrangements across different species and during cell differentiation. In addition to kDNA, the kinetoplast contains multiple proteins, including those involved in mitochondrial DNA topology and metabolism, such as the kinetoplast-associated proteins (KAPs). In this work, we obtained mutant cells to investigates the role of KAP7 in two trypanosomatid species, *Trypanosoma cruzi* and *Angomonas deanei*, which have distinct kinetoplast shapes and kDNA arrangements.

**Methods:**

For this purpose, the kDNA replication process and cell morphology and ultrastructure were evaluated using microscopy methods. Furthermore, the proliferation of cells treated with genotoxic agents, such as cisplatin and ultraviolet radiation, was analyzed.

**Results:**

In *A. deanei*, which contains a symbiotic bacterium, KAP7 seems to be essential, since the deletion of one *KAP7* allele generated mutants with a decay in cell proliferation, as well as changes in kDNA structure and replication. In *T. cruzi*, null mutants exhibited disturbances in kDNA replication, although the overall topology remained unaltered. The use of cisplatin and ultraviolet (UV) radiation affected the ultrastructure of *A. deanei* and *T. cruzi*. Cisplatin promoted increased kDNA compaction in both *KAP7* mutants, but only in *T. cruzi* did the proliferative capacity fail to recover after treatment, as was also observed following UV radiation exposure.

**Conclusions:**

Proteins associated with DNA are evolutionarily conserved and usually perform similar functions in different organisms. Our findings reveal that KAP7 is involved in kDNA replication, but its roles differ in trypanosomatid species: in *A. deanei*, KAP7 is associated with kDNA arrangement, while in *T. cruzi*, it is related to mitochondrial metabolism, such as kDNA replication and damage response.

**Graphical Abstract:**

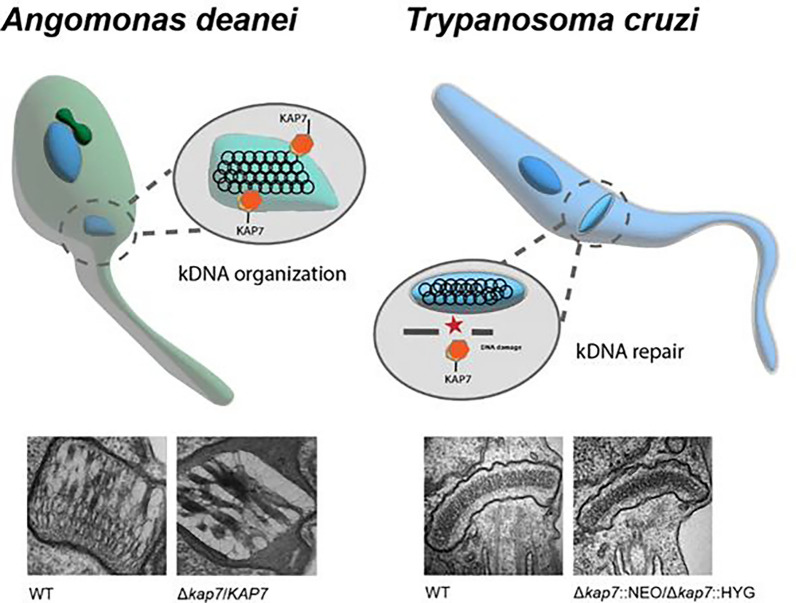

**Supplementary Information:**

The online version contains supplementary material available at 10.1186/s13071-025-06985-8.

## Background

Trypanosomatid kinetoplast DNA (kDNA) is contained in the mitochondrial matrix, consisting of thousands of minicircles (ranging from 0.5 to 10 kb) and approximately 25 maxicircles (ranging from 20 to 40 kb) that are interlocked, forming an intricate network. Maxicircles encode rRNAs and several proteins involved in energy transduction, as observed in other eukaryotes. Transcripts generated from maxicircles are highly edited in reactions that involve the addition or removal of uridylate residues. A large number of minicircles encode different types of small guide RNAs that are templates for maxicircle editing, thus generating functional mitochondrial messenger RNAs (mRNAs) [1, 2].

DNA replication is a complex and unique process that involves multiple enzymes. Minicircles are cleaved by mitochondrial topoisomerase II and released into the kinetoflagellar zone (KFZ), the region between kDNA and the inner mitochondrial membrane that faces the flagellar basal body. Following replication in the KFZ, minicircles migrate to the antipodal sites, where primers are removed, and gaps between Okazaki fragments are repaired. At these sites, minicircles are reconnected to the network periphery by topoisomerase II and remain open with a single nick, until all of them are duplicated, ensuring that each molecule replicates only once per cell cycle [3, 4]. The positioning of the network in the mitochondrial matrix and the segregation of daughter networks are facilitated by the tripartite attachment complex (TAC). This complex is composed of filaments that link the basal body with the outer mitochondrial membrane and also connect the inner mitochondrial membrane to kDNA. Upon duplication, the kDNA network is cleaved as basal bodies move apart [5–7].

Unlike most eukaryotes, the mitochondrial DNA replication of trypanosomatids is regulated during the cell cycle, with kDNA synthesis starting just before the nuclear S phase, followed by kDNA excision and kinetoplast division during the G2 phase of the cell cycle [2]. Kinetoplast biogenesis occurs in five steps: (i) kDNA synthesis, (ii) selection of the excision plane, (iii) the excision itself, when the kDNA cleaves into two networks, (iv) the separation of the networks and (v) the kinetoplast partitioning between daughter cells during cytokinesis [8]. Two distinct mechanisms for organizing minicircles in the network postreplication have been described: in *Trypanosoma cruzi* and most trypanosomatids, the kDNA network rotates relative to the antipodal sites (ring mechanism). In *T. brucei*, newly replicated minicircles accumulate at the antipodal sites, where they are reattached, leading to a shrinking of the network’s central zone (polar mechanism) [9]. Recently, a new proposal has emerged to explain kDNA replication in *T. brucei*, which considers that minicircle duplication occurs simultaneously at the two antipodal sites, where replication is initiated and proceeds via theta intermediates [10]. Although maxicircle replication remains less understood, it is also unidirectional, with the maxicircles remaining attached to the kDNA network throughout the process, which is regulated by a DNA helicase [11–13].

The organization of kDNA varies among species and developmental stages. In most trypanosomatids, such as *Crithidia fasciculata*, *Leishmania amazonensis*, *T. brucei*, as well as in the epimastigote and amastigote replicative forms of *T. cruzi*, kDNA fibrils are tightly packed. Conversely, species such as *Angomonas deanei*, which harbors an endosymbiont, and the trypomastigote form of *T. cruzi*, exhibit a looser kDNA arrangement that fills the entire kinetoplast matrix [14–17]. Despite presenting variations in genetic sequences, the physical properties of kDNA networks are largely conserved in trypanosomatids and allow the formation of intriguing networks composed of concatenated circles [18].

The kDNA arrangement has been related to the activity of kinetoplast-associated proteins (KAPs). Such small basic proteins are described as histone H1-like and also as nonhistone proteins that contain the high mobility group (HMG) box domain that binds DNA. Most mitochondrial proteins, as KAPs, have an N-terminal targeting signal, but several of them enter the matrix through a C-terminal targeting peptide, such as topoisomerase II, localized at the antipodal sites [19–21]. Similar to the basic proteins associated with nuclear DNA, KAPs neutralize the negative charges of kDNA, playing pivotal roles in its topology, as well as in mitochondrial DNA duplication and transcription [22–24].

Biochemical and ultrastructural analyses showed that basic proteins associated to the kDNA are differentially expressed during the developmental stages of *T. cruzi* [25, 26]. The expression of KAPs 4 and 6 was detected in all phases of the parasite life cycle. However, in epimastigotes and amastigotes, such proteins were distributed throughout the kinetoplast DNA, while in trypomastigotes and intermediate forms, they were present only at the periphery of the kDNA network [27]. Deletion of *KAP3* by homologous recombination did not promote changes in proliferation, differentiation, kDNA topology, or infectivity, indicating that different KAPs may have overlapped functions [28].

Phylogenetic analyses revealed an expanded repertoire of KAPs in symbiont-harboring trypanosomatids and indicated that sequences for KAP 4 and 7 are conserved and present in all species of the Trypanosomatidae family so far investigated [29]. CRISPR–Cas9-mediated deletion of *KAP4* revealed its involvement in kDNA arrangement and replication in *A. deanei* but not in kDNA repair. Mutant cells exhibited decreased proliferation and ultrastructural changes, as well as loss of the symbiotic bacterium, suggesting coordinated division between the prokaryote and kinetoplast replication [30]. More recently, it was shown that *KAP7* is essential for *A. deanei* but not for *T. cruzi.* However, in both species, this protein is involved in ultraviolet (UV) and cisplatin kDNA damage response [31].

Several studies have investigated trypanosomatid kDNA metabolism after lesions caused by genotoxic agents. Evidence suggests that *T. cruzi* can repair oxidative damage to kDNA, with DNA glycosylases participating in mtDNA damage responses [32–34]. Ultraviolet radiation type C (UV-C) and cisplatin can induce damage to kDNA molecules, providing means to investigate this hypothesis. UV-C exposure creates photoproducts by forming covalent bonds between adjacent pyrimidines and DNA lesions, notably cyclobutane dimers (CPD) and the 6–4 photoproduct [35, 36]. Cisplatin interacts with the nucleophilic centers of DNA and proteins, blocking transcription and cell replication [37, 38].

KAP proteins are crucial for maintaining kDNA organization and metabolism in trypanosomatids, processes essential for cell proliferation. However, their specific involvement in kDNA replication and repair remain to be elucidated. In this study, we investigated the effects of *KAP7* gene deletion on proliferation, cellular ultrastructure, and kDNA replication in two trypanosomatid species: *T. cruzi*, the causative agent of Chagas disease, and *A. deanei*, a nonpathogenic parasite harboring symbiotic bacteria. Additionally, we assessed the mutant response to genotoxic agents, such as cisplatin and ultraviolet radiation, both known to induce mitochondrial DNA damage. These species were selected owing to their distinct life cycles, kinetoplast shapes, kDNA arrangements, and the essentiality of *KAP7* in each. Our findings suggest that *KAP7* function is species-specific and may vary among species with different kDNA topology.

## Methods

### Cell culture

*Angomonas deanei* wild type strain (WT-ATCC 30255) was grown in Warren medium [39] supplemented with 10% fetal bovine serum. Culture maintenance was carried out through weekly passages inoculating 10% of an established cell culture in fresh medium. Wild-type (WT) and T7RNAPol-SpCas9 (T7 Cas9) cell lines were cultured at 28 ˚C for 24 h, as previously described [30]. We were unable to generate KAP7-null mutant cells in *A. deanei*, only a heterozygous *KAP7* mutant (Δ*kap7*/*KAP7*), as described by Repolês et al. [31]. Cells used in experimental tests were in the exponential growth phase, which is equivalent to 24–48 h of cultivation. After this period, part of the culture was stored for 1 week at 4 °C until the next passage.

Epimastigote forms of the *Trypanosoma cruzi* clone Dm28c (Contreras et al., [66, 67]), including both the WT strain and the *KAP7*-null mutants (Δ*kap7*), described by Repolês et al. [31], were cultivated in liver infusion tryptose (LIT medium) [40] at 28 °C. Passages were carried out every 4 days at a ratio of 1:10. Cells used in various assays were harvested during the logarithmic growth phase (72–96 h).

### Analysis of cell proliferation

Proliferation assays started with an initial cell concentration of 1.0 × 10^6^ cells/mL and assessed every 24 h up to 72 h. Cell density was determined by counting live protozoa using a flow cytometry-based method, with cell size determined by detection of forward side scatter (FSC) on SSA detector in BD Accuri C6 flow cytometer (Becton Dickinson Bioscience BDB, San Jose, CA, USA). The relative growth rate (*μ*) was established during the exponential growth phase using the exponential function *y* = *Ae*^*Bx*^, where *A* is the initial concentration at *x* = 0, e is Euler’s number, *B* (also called *μ*) is the growth or decay rate, *x* is time, and *y* is the cell concentration at time *x*. This estimation was based on the relationship between culture cell density (cells/mL) and culture time (hours) for each strain. Graphs were generated using data collected from up to 48 h of growth, which represents the exponential phase in which all assays were conducted. The cell duplication time (DT) was calculated using the formula DT = ln2 / *µ*. The cell concentration was measured 24 h after subculturing for *T. cruzi* and 48 h after subculturing for *A. deanei*, and reductions were calculated in comparison with the respective WT strain.

### Genotoxic treatment

WT and *KAP7*-mutant cells of *A. deanei* and *T. cruzi* were plated at a density of 1.0 × 10^7^ cells/mL and were exposed or not (control group) to genotoxic agents. For cisplatin treatment, cells were incubated with 300 μM of the drug for 1 h, washed thrice with phosphate-buffered saline (PBS) (pH 7.2) and resuspended in fresh medium. UV-C irradiation was carried out using a germicidal lamp at a fluence rate of 1,500 µ J/cm^2^ (GS GeneLinker UV Chamber, Bio-Rad). For proliferation analysis, the number of surviving cells after cisplatin treatment was determined at 0 h (just before the treatment) up to 72 h for *A. deanei* or 144 h for *T. cruzi*, considering their different generation times (6 h and 24 h, respectively). Cell proliferation after UV irradiation was also evaluated, with the number of surviving cells counted from 0 h up to 24 h for *A. deanei* or 120 h for *T. cruzi*. Erythrosine, a vital stain, was used to differentiate living and dead cells during quantification using a Neubauer counting chamber. Experiments were performed in biological triplicate.

### Fluorescence microscopy

Protozoa were collected by centrifugation at 2,000*g*, washed once with PBS pH 7.4, fixed in 4% paraformaldehyde in the same solution and mounted on poly-l-lysine-coated microscope coverslips. To analyze the symbiont shape, *A. deanei* was incubated in a blocking solution containing 1.5% bovine serum albumin (BSA) and 0.02% Tween 20 diluted in PBS pH 8.0. Then, slides were incubated for 1 h with an antibody produced against the symbiont porin [41] diluted in blocking solution (1:10). Thereafter, cells were washed with PBS and incubated for 45 min with Alexa 488-conjugated anti-mouse immunoglobulin (Ig)G (Molecular Probes, USA) diluted 1:200 in blocking solution. To determine cellular patterns of DNA-containing structures, such as the nucleus, kinetoplast, and symbiont, slides containing *A. deanei* or *T. cruzi* were washed with PBS and incubated with 10 μg/ml 4′,6-diamidino-2-phenylindole (DAPI, from Molecular Probes) for 10 min. After washing with PBS, slides were mounted using ProLong Gold (Molecular Probes) and analyzed using an Elyra PS.1 microscope (Zeiss, Germany). Analyses were based on counts of 1,000 cells of WT and *KAP7* mutants.

### In situ labeling of kDNA networks

After washing, cells were fixed in 2% paraformaldehyde diluted in PBS, pH 7.2, for 5 min. Then, cells were adhered to slides coated with poly-l-lysine-coated for 10 min and washed twice in PBS containing 0.1 M glycine for 5 min. In the next step, samples were permeabilized in methanol for 1 h at 20 °C and rehydrated thrice in PBS washes during 5 min. After this period, cells were incubated for 60 min at room temperature in 25 μL of reaction solution containing terminal deoxynucleotidyl transferase (TdT) reaction buffer (Roche Applied Science), 2.0 mM CoCl_2_, 10 μM deoxyadenosine triphosphate (dATP), 2.5 μM Alexa Fluor 488-deoxyuridine triphosphate (dUTP) (Molecular Probes), and 10 units of TdT (Roche Applied Science). The reaction was stopped with three washes in 2× saline-sodium citrate (SSC) buffer for 5 min. Samples were mounted in slides using the anti-fading reagent ProLong Gold containing 5 μg/mL DAPI and then were observed on an Axiobserver microscope (Carl Zeiss). Images were collected, and analyses were based on counts of 1,000 cells of WT and KAP7 mutants considering the kDNA replication as previously described by Liu and Englund (2007) [42].

### Scanning electron microscopy (SEM)

Protozoa were fixed for 1 h in 2.5% glutaraldehyde II (Sigma, USA) diluted in 0.1 M cacodylate buffer pH 7.2. Then, cells were then adhered to glass coverslips precoated with poly-l-lysine, postfixed for 1 h with 1% osmium tetroxide diluted in cacodylate buffer, and dehydrated in a graded alcohol series (50%, 70%, 90%, and two exchanges of 100% ethanol for 10 min each step). Samples were critical-point dried in a Leica EM CPD030 apparatus (Leica, Germany). Specimens were sputtered with gold in a Balzers FL9496 unit (Postfach 1000 FL-9496, Balzers, Liechtenstein) and observed in an EVO 40 VP SEM (Zeiss, Germany). In all assays, approximately 500 cells were observed.

### Transmission electron microscopy (TEM)

Protozoa were fixed for 1 h in 2.5% glutaraldehyde diluted in 0.1 M cacodylate buffer, pH 7.2. Then, samples were washed twice in cacodylate buffer and postfixed in a solution containing 1% osmium tetroxide, 0.8% potassium ferrocyanide, and 5 mM calcium chloride diluted in 0.1 M cacodylate buffer, for 1 h. Samples were then washed in cacodylate buffer, dehydrated in a graded series of acetone solutions (50%, 70%, 90%, and two exchanges of 100% acetone for 10 min each step) and embedded in Polybed resin. Ultrathin sections were stained with 5% uranyl acetate for 45 min and lead citrate for 5 min before observation in a Tecnai™ Spirit TEM transmission electron microscope (Zeiss, Oberkochen, Germany). In all assays performed, approximately 500 cells were observed.

## Results

To investigate the cellular response to genotoxic stress in *A. deanei and T. cruzi*, previously generated *KAP7 *mutants were used [31]. Prior to assessing their growth, ultrastructure, and susceptibility to DNA-damaging agents, we confirmed the genetic modifications by PCR and evaluated their proliferation under standard culture conditions (Table 1, Additional file 1: Fig. S1, Additional file 3: Table S1).

**Table 1:** Comparison of the data obtained with the proliferation curves of WT and *KAP7* mutants of *A. deanei* (48 h) and *T. cruzi* (72 h)

	Ad WT	Ad Δ*kap7*/*KAP7*	Tc WT Dm28c	Tc Δkap::NEO/ Δ*kap7*::HYG
Proliferation rate (*μ*) in log phase	2.33 ± 0.08	1.65 ± 0.12	0.69 ± 0.11	0.62 ± 0.03
*μ* reduction in relation to WT	–	29%	–	10%
Duplicate time (h)	7.14 ± 0.26	9.62 ± 0.31	25.18 ± 3.92	28.10 ± 1.47
Cell concentration reduction in relation to WT	–	70%	–	28%

**Fig. 1:**
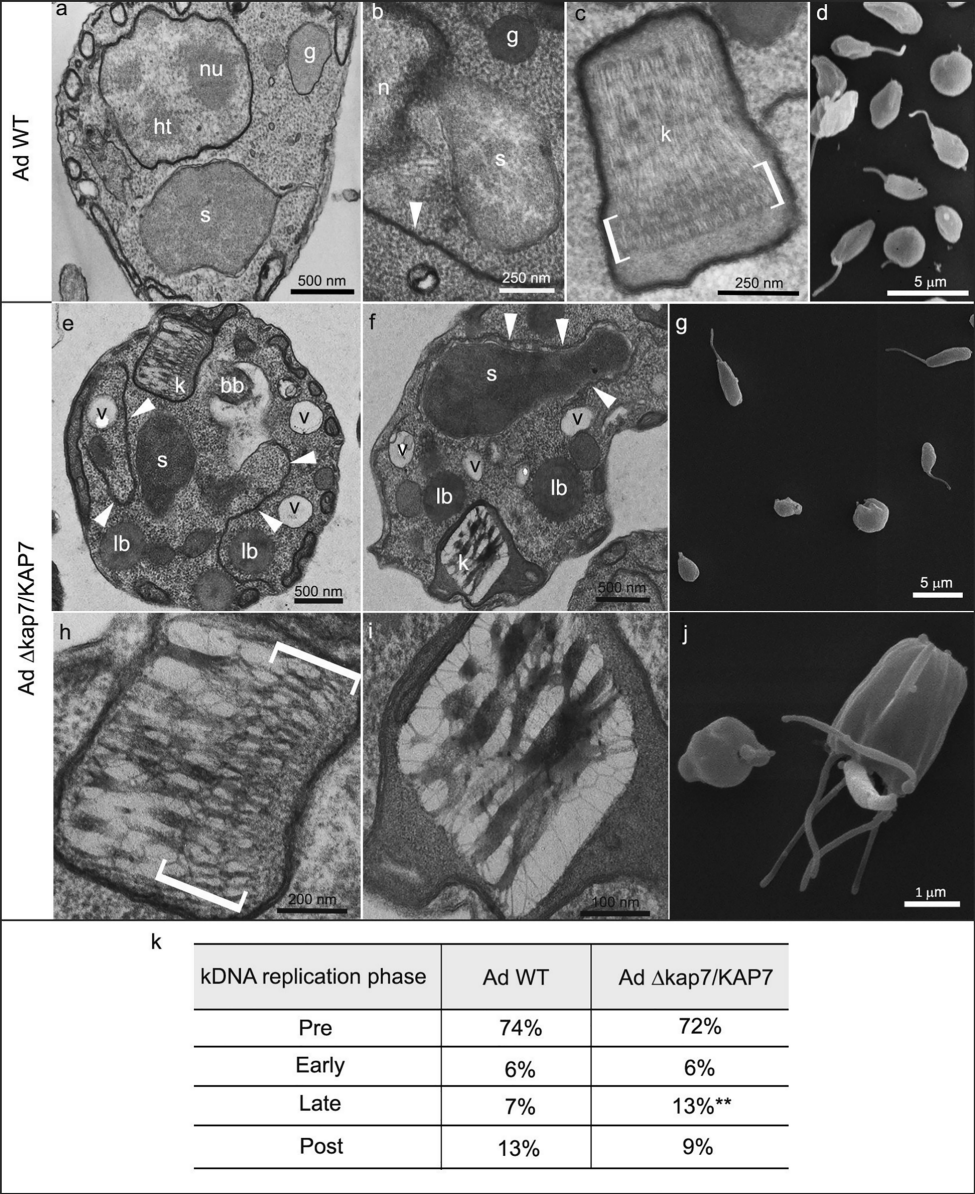
Ultrastructure and morphology of *A. deanei* WT and *KAP7* mutants (AdΔ*kap7*/*KAP7*). **a**–**d**, WT cells. **d**–**j**, *KAP7* mutant. The square brackets indicate the region where the kDNA is more densely packed and the white arrowhead the endoplasmic reticulum profiles. **k**, Quantification of cells in different phases of kDNA replication. A total of 900 WT and *KAP7* mutant cells were counted in 3 independent experiments. To compare control and mutant cells, an unpaired *t*-test was performed (**P < 0.01). *bb* basal body, *g* glycosome, *ht* heterochromatin, *k* kinetoplast DNA, *lb* lipid body, *nu* nucleolus, *s* symbiont, *v* vacuole

Transmission electron microscopy images (TEM) obtained after 24 h of cultivation showed that, in *A. deanei* WT cells, the nucleus occupies a central position in the cell body, containing a nucleolus surrounded by heterochromatin, which is also observed in the nuclear periphery (Fig. 1a). The symbiont was usually observed close to the nucleus and delimited by two membranes (Fig. 1a–b). The kinetoplast has a trapezoid shape and contains kDNA fibrils with different degrees of compaction: in the central region, the kDNA is loosely arranged, while in the region facing the basal body (indicated by square brackets), a more compact arrangement is observed (Fig. 1c). Using scanning electron microscopy images (SEM), it was possible to visualize the classic choanomastigote shape, which has smooth surfaces (Fig. 1d).

The ultrastructure analysis of *A. deanei* Δ*kap7*/*KAP7* cells showed extensive endoplasmic reticulum (ER) profiles, especially surrounding the symbiont (Fig. 1e–f, arrowheads). In such protozoa, the kinetoplast maintained the trapezoid shape but presented increasing degrees of kDNA compaction (Fig. 1h–i and Additional file 2: Fig. S2). By SEM, *A. deanei* mutants displayed different shapes when compared with WT cells (Fig. 1d), presenting atypical shortened and rounded formats, as well as misshapen cell body with multiple flagella, an indication of cytokinesis impairment (Fig. 1g, j and additional file 2: Fig. S2). Indeed, dUTP incorporation by the deoxynucleotidyl transferase terminal (TdT) indicated that kDNA replication was affected in *A. deanei* Δ*kap7*/*KAP7*, with the percentage of cells in the late phase being almost twice as high in mutants when compared with the wild-type strain (Fig. 1k).

**Fig. 2:**
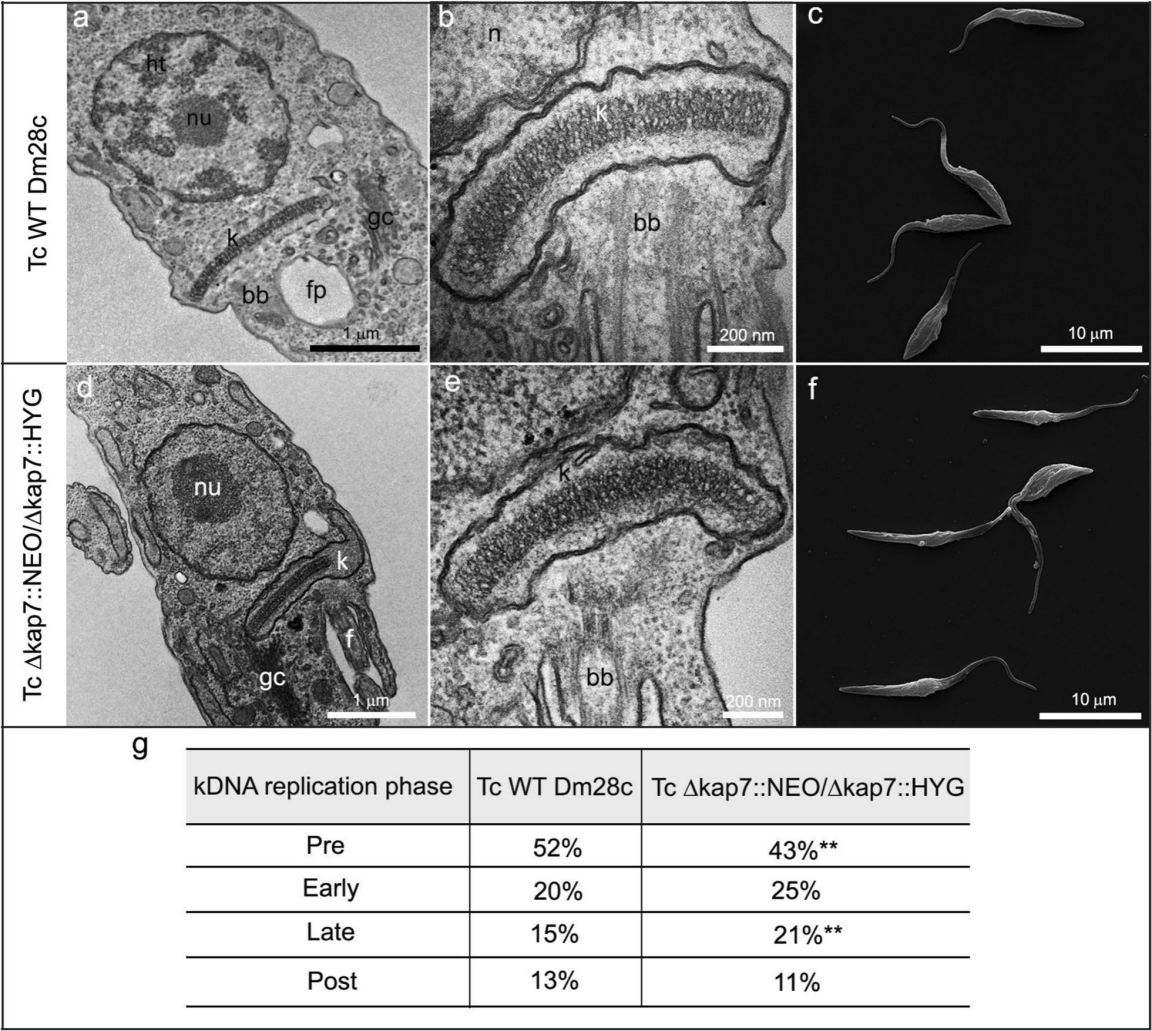
Ultrastructure and morphology of *T. cruzi* WT and *KAP7*-mutant strains (TcΔkap7::NEO/Δ*kap7*::HYG). **a–c**, WT cells, **d**–**f**, *KAP7 *mutant cells. **a**,**b**,**d**,**e**, cells observed by TEM; **c**,**f**, cells observed by SEM. **g** Quantification of cells in different phases of kDNA replication. A total of 900 WT and *KAP7*-mutant cells were counted in three independent experiments. To compare control and mutant cells, an unpaired *t*-test was performed (**P < 0.01). *bb* basal body, *f* flagellum, *fp* flagellar pocket, *gc* Golgi complex, *ht* heterochromatin, *k* kinetoplast, *n* nucleus, *nu* nucleolus

*T. cruzi* lacking both *KAP7* alleles did not exhibit cell ultrastructure changes compared with WT cells. TEM images revealed the nucleus centrally positioned in both strains after 24 h of culture (Fig. 2a, d), with disc-shaped kinetoplast containing tightly packed kDNA fibrils (Fig. 2b, e). The SEM revealed characteristic epimastigote morphology in both WT and mutant strains (Fig. 2c, f). Analysis of kDNA replication showed a lower percentage of cells in the pre-replication stage and an increase in the late replication phase compared with WT protozoa (Fig. 2g).

**Fig. 3:**
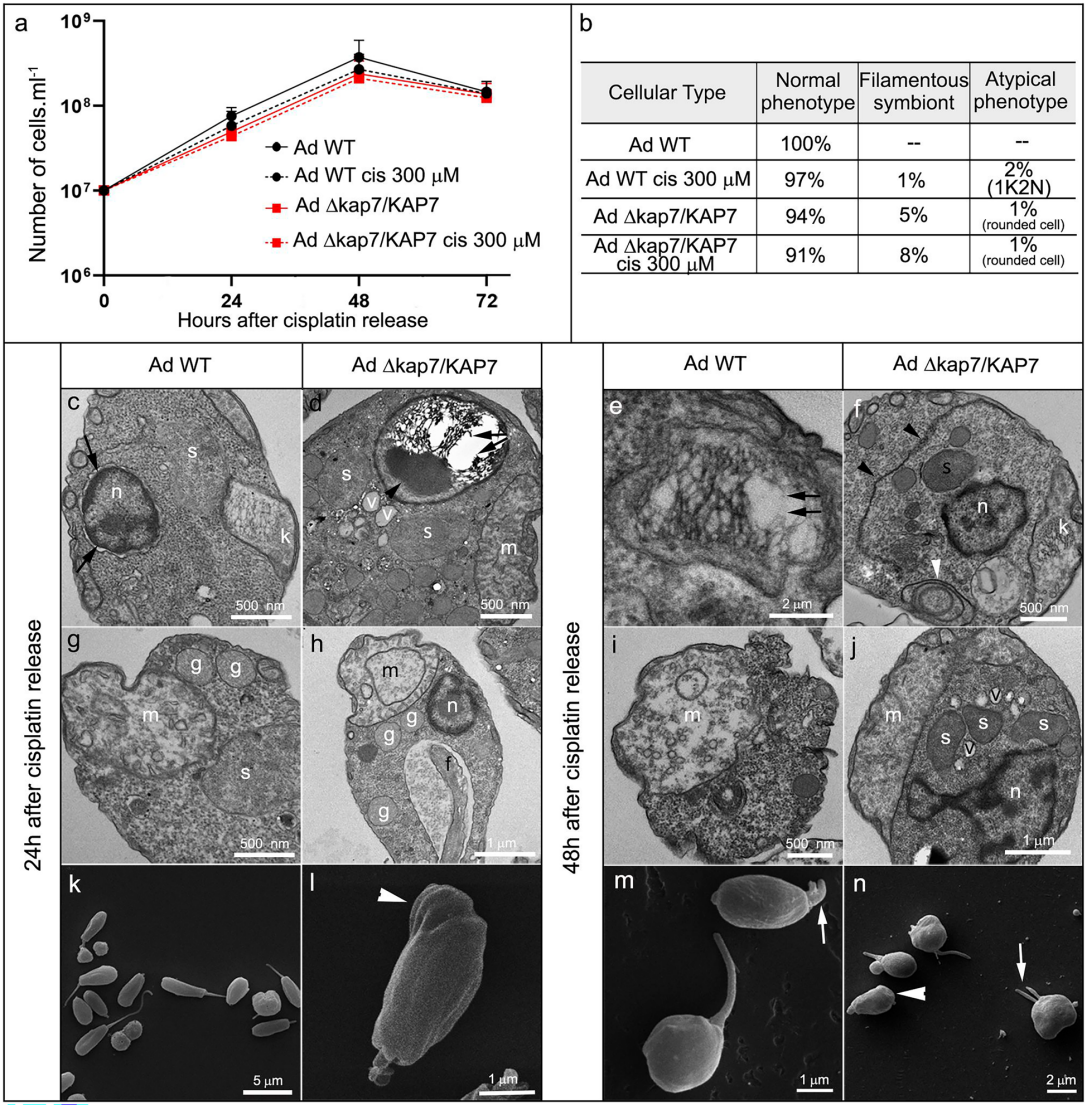
Effects of 300 μM of cisplatin on cell proliferation (**a**) and cellular patterns (**b**) of *A. deanei* WT and *KAP7*-mutant cells (AdΔ*kap7*/*KAP7*). For quantification of cellular patterns, 1,000 cells of each strain were counted, treated or not treated with the inhibitor. The morphology and ultrastructure of WT and *KAP7* - mutant cells of *A. deanei* were also analyzed by TEM: (**c**, **g** and **e**, **i**) WT cells, (**d**, **h**, and **f**, **j**) *KAP7*-mutant cells, or by SEM: (**k**–**l**) WT cells, (**m**–**n**) *KAP7* mutants. In (**c**), black arrows indicate detachment of the nuclear membranes; in (**d**), black arrowheads point to atypical electron density in the kinetoflagellar region; in (**d–e**), black arrows indicate ruptured kDNA fibers and in (**f**) ER profiles. In (**l**), white arrowheads indicate a protozoan with protrusion in the posterior region and in (**n**) rounded cells. In (**m**) and (**n**) white arrows point to two short flagella. *g* glycosome, *k* kinetoplast, *m* mitochondrion, *n* nucleus, *s* symbiont, *v* vacuole

Next, we exposed both WT and *KAP7 *strains of *T. cruzi* and *A. deanei* to cisplatin and ultraviolet radiation (UV-C) to evaluate the potential involvement of *KAP7* in kDNA metabolism. In *A. deanei*, WT cells showed a slight reduction in proliferation after cisplatin treatment, while no such effect was observed when comparing treated and untreated *A. deanei* Δ*kap7*/*KAP7* (Fig. 3a). Treatment of WT and Δ*kap7*/*KAP7* cells with cisplatin had minimal impact on the number and distribution of DNA-containing structures, although in WT protozoa, a small increase of cells completing mitosis before the kinetoplast division (1K2N) was observed, a phenomenon that was absent in untreated WT cells. In *A. deanei* Δ*kap7*/*KAP7*, cisplatin slightly elevated the number of cells with filamentous symbionts (Fig. 3b).

After 24 h of cisplatin release, *A. deanei* WT cells did not exhibit changes in kDNA arrangement but presented detachment of the nuclear membranes (Fig. 3c) and swelling of mitochondrial branches (Fig. 3g). In *A. deanei* Δ*kap7*/*KAP7*, treatment induced kDNA disruption of fibrils (Fig. 3d, black arrow), which was evidenced by the increase of electron lucent regions in the kinetoplast and by the appearance of an atypical electron density area in the kinetoflagellar region (Fig. 3d, black arrowhead). Mitochondrial swelling and enlargement of cristae was also observed (Fig. 3h). SEM images showed varied morphologies in both WT and Δ*kap7*/*KAP7* protozoa after treatment. WT cells exhibited smaller and rounded shapes (Fig. 3k), as also observed in Δ*kap7*/*KAP7*, which rarely displayed protrusions in the posterior region of the cell body (Fig. 3l, white arrowhead).

After 48 h of cisplatin release, *A. deanei* WT cells displayed ruptured kDNA fibers and mitochondrial swelling (Fig. 3e, black arrow). Δ*kap7*/*KAP7* exhibited extensive ER profiles, especially near the symbiont, which was seen surrounded by vacuoles (Fig. 3f, j). Additionally, myelin figures (Fig. 3f, white arrowhead) and enlargement of mitochondrial cristae (Fig. 3j) were observed in these cells. The prolonged treatment of WT and Δ*kap7*/*KAP7* cells led to flagellum shortening in rounded cells, along with the emergence of cells displaying two short flagella (Fig. 3m, n, arrows). In mutant cells, protozoa lacking the flagellum were also observed (Fig. 3n, arrowhead).

**Fig. 4:**
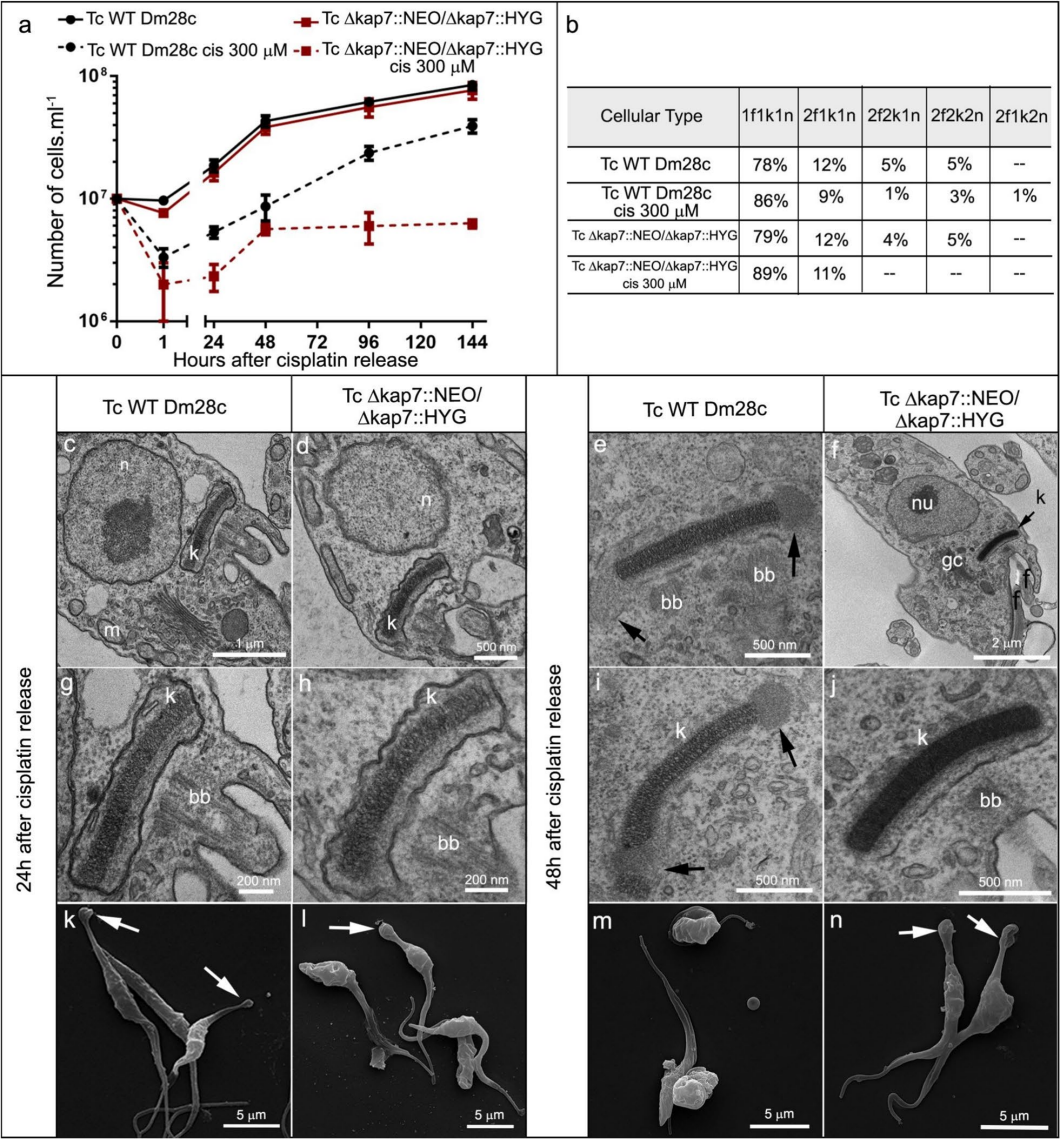
Effects of 300 μM of cisplatin on cell proliferation (**a**) and cellular patterns (**b**) of *T. cruzi* WT or *KAP7* mutants (TcΔ*kap7*::NEO/Δ*kap7*::HYG). For quantification of cellular patterns, 1,000 cells of each strain were counted, treated or not treated with the inhibitor. The morphology and ultrastructure of WT and *KAP7* mutant cells of *T. cruzi* after 24 h and 48 h of treatment were also analyzed by TEM: (**c**, **g** and **e**, **i**) WT cells, (**d**, **h** and **f**, **j**) *KAP7*mutant cells, or by SEM: (**k**–**l**) WT cells, (**m**–**n**) *KAP7* mutants. Black arrows indicate antipodal sites, and white arrows enlargement of the posterior end of the cell body. *bb* basal body, *gc* Golgi complex, *k* kinetoplast, *m* mitochondrion, *n* nucleus, *nu* nucleolus

Untreated *T. cruzi* WT and Δ*kap7*::NEO/Δ*kap7*::HYG strains exhibited similar proliferation rates. After incubation with cisplatin, both cell types showed reduced growth, but only WT cells progressively recovered proliferation after drug release. The mutant strain initially showed recovery in proliferation, followed by an early entry into the stationary phase (Fig. 4a). However, cisplatin treatment did not induce a significant increase of abnormal phenotypes, considering the number of DNA-containing structures (Fig. 4b). Although no changes in kDNA topology were observed in WT (Fig. 4c, g) or in *KAP7* mutants after 24 h of cisplatin release, (Fig. 4d, h), both cell types showed nuclear DNA unpacking (Fig. 4c, d). After 48 h of cisplatin release, the appearance of electron lucent regions at the distal ends of the kDNA disk was observed, which may correspond to antipodal sites but more likely represent the onset of kDNA network disorganization due to the genotoxic effects of cisplatin (Fig. 4e, i, black arrows). In *KAP7* mutants, an increased electron density of the kDNA was observed, indicating greater compaction of the network (Fig. 4f, j). SEM morphological analysis revealed that treatment with cisplatin led to a subtle enlargement of the posterior end of the cell body in 5% of both strains (Fig. 4k–n, white arrows).

**Fig. 5:**
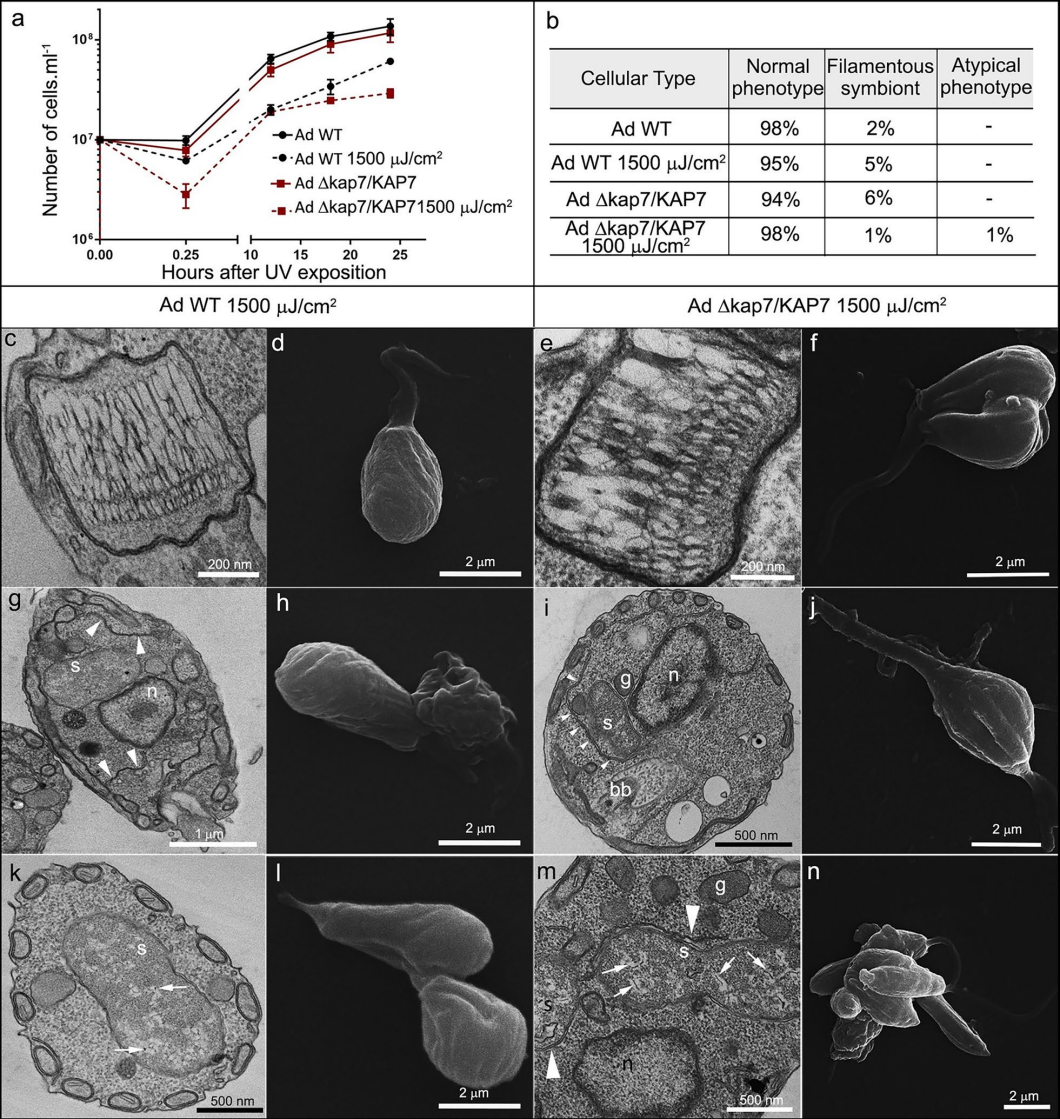
Effects of UV-C irradiation on cell proliferation (**a**) and cellular patterns (**b**) of *A. deanei* WT or *KAP7* mutants (Ad Δ*kap7*/*KAP7*). Considering the quantification of cellular patterns, 700 cells of each strain were counted, with exposure or without exposure to UV. Effects of UV-C radiation on the morphology and ultrastructure of WT and *KAP7* mutant cells of *A. deanei* were also evaluated by TEM: (**c**, **g**, **k**) WT cells, (**e**, **i**, **m**) *KAP7*-mutant cells, or by SEM: (**d**, **h**, **l**) WT cells, (**f**, **j**, **n**) *KAP7* mutants. White arrows indicate DNA compaction of the symbiont and white arrowheads ER surrounding the symbiont. *bb* basal body, *g* glycosome, *n* nucleus, *s* symbiont

UV-C radiation had a more pronounced impact on the survival of *A. deanei* Δ*kap7*/*KAP7* compared with WT cells. Although proliferation resumed for both strains, neither reached the same cell density as the nonirradiated controls after 24 h (Fig. 5a). Analysis and quantification of DNA-containing structures revealed increase of filamentous symbionts in irradiated WT cells (from 2 to 5%). The lower number of filamentous symbionts in mutant cells after irradiation may be related to bacterium lysis (from 5% to 1%). A small percentual of atypical morphotypes (1%) was observed in WT and mutant cells after UV irradiation. These phenotypes included rounded cells and protozoa with multiple cell bodies attached (Fig. 5b).

After 24 h of UV-C exposure, WT *A. deanei* cells showed no changes in kDNA arrangement compared with untreated protozoa (Fig. 5c). Conversely, *KAP7* -mutant cells exhibited higher kDNA compaction, which was evidenced by the electron-lucent areas between DNA fibers (Fig. 5e). The unpacking of nuclear DNA was seen in WT and mutant cells after UV exposure (Fig. 5g, i, m), whereas the symbiont DNA showed intensive compaction (Fig. 5k, m, white arrows). The symbiotic bacterium was frequently seen surrounded by ER profiles (Fig. 5g, i, m, white arrowheads). SEM data indicated morphological changes induced by UV in both WT (Fig. 5d, h, l) and mutant strains (Fig. 5f, j, n), with enlarged cell bodies and, in some instances, presenting multiple bodies, thus suggesting cytokinesis arrest.

**Fig. 6:**
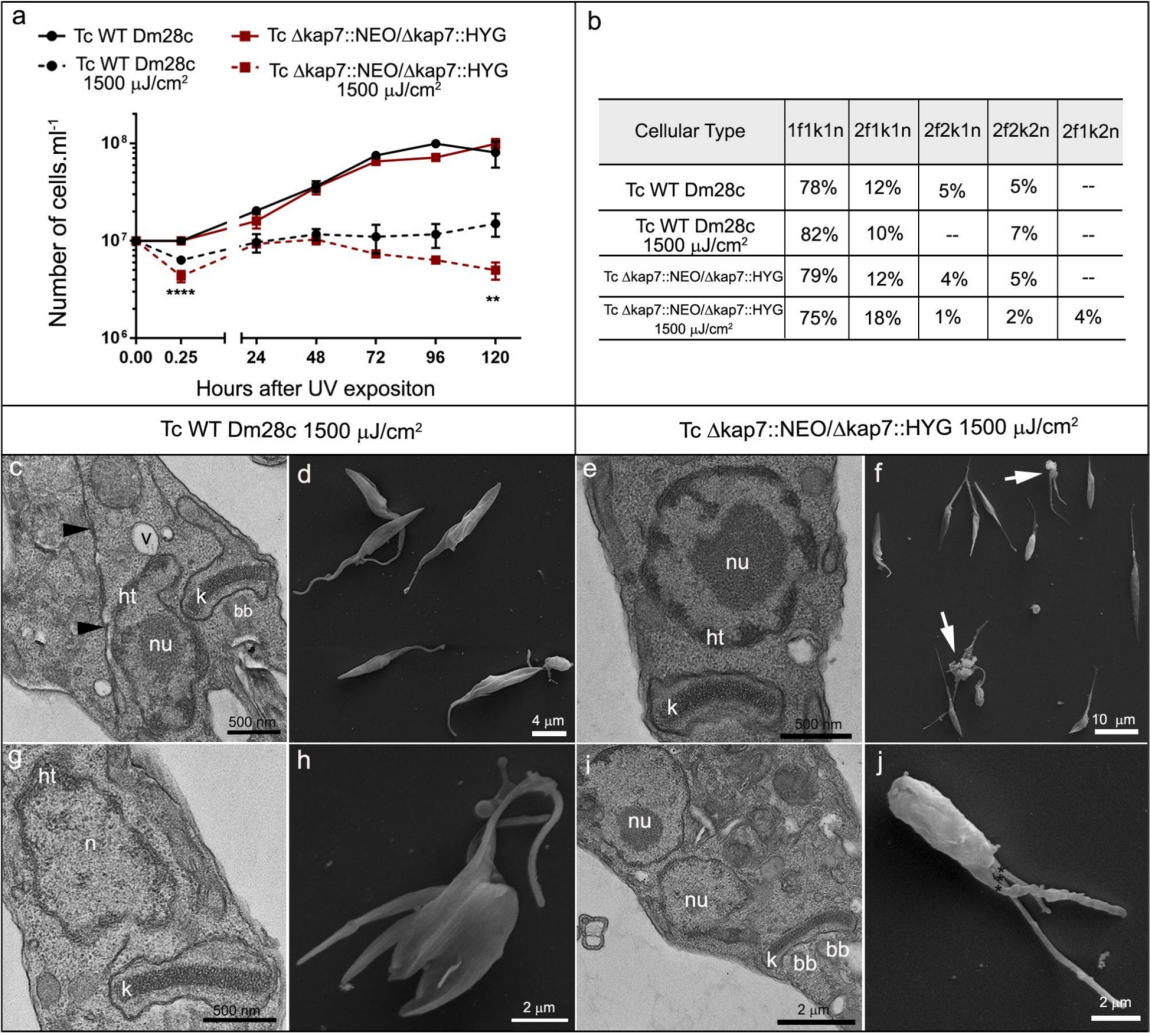
Effects of UV-C irradiation on cell proliferation (**a**) and cellular patterns (**b**) of *T. cruzi* WT or *KAP7* mutants (TcΔ*kap7*::NEO/Δ*kap7*::HYG). Considering the quantification of cellular patterns, 700 cells of each strain were counted, exposure or not exposure to UV. Effects of UV-C radiation on the morphology and ultrastructure of WT and *KAP7* mutant cells of *T. cruzi* were also registered by TEM: (**c**, **g**) WT cells, (**e**, **i**) *KAP7* mutant cells, or by SEM: (**d**, **h**) wild-type (WT) cells, (**f**, **j**) *KAP7* mutants. White arrows indicate interconnected cell bodies and black arrowheads ER profile. *bb* basal body, *ht* heterochromatin, *K* kinetoplast, *n* nucleus, *nu* nucleolus, *v* vacuole

Proliferation of both *T. cruzi* WT and Δ*kap7*::NEO/Δ*kap7*::HYG strains remained constant after irradiation, although the initial number of cells was significantly affected by UV irradiation. When the mutant strain was exposed to UV, cells were more sensitive after 15 min of UV exposure, and the cell count remained unchanged between 24 and 72 h postirradiation. After that, a notable reduction, compared with that of nonirradiated mutant cells, was observed (Fig. 6a). Similarly to cisplatin treatment, quantitative analysis of morphotypes in both strains, whether exposed to UV or not, revealed that the majority of the cells exhibited one nucleus, one kinetoplast, and one flagellum. After irradiation, only a small percentage of *KAP7 *mutants (4%) displayed an atypical cell pattern with two nuclei, two flagella, but a single kinetoplast, an indication of division impairment (Fig. 6b).

The typical ultrastructure of *T. cruzi*, both for WT (Fig. 6c, g) and *KAP7* mutant cells (Fig. 6e, i), was largely preserved, although nuclear heterochromatin unpacking was observed (Fig. 6g, i), as well as an increase in ER profiles on irradiated WT cells (Fig. 6c, black arrowheads). Although morphology was generally preserved in most protozoa of both strains, cells rarely showed multiple flagella or interconnected cell bodies (Fig. 6h, f, j, arrows), suggesting impaired cytokinesis.

## Discussion

The kinetoplast is the portion of the single trypanosomatid mitochondrion that contains the kDNA, a network composed of interlocked circles packed by kinetoplast-associated proteins (KAPs). KAPs are highly basic, neutralizing DNA strands, thus facilitating the binding of proteins that participate in essential processes, such as kDNA replication, transcription, as well as RNA processing and translation [2, 43]. Genes encoding *KAP7 *are found in all trypanosomatid species, including those from *Leishmania* and *Trypanosoma* genera, as well as in monoxenous species as *Crithidia*, *Leptomonas*, and *Angomonas*. This widespread distribution highlights the importance of this protein in kDNA organization and metabolism [29–31, 44].

The single deletion of *A. deanei KAP7* allele led to increased kDNA compaction, reinforcing the involvement of KAPs in mitochondrial DNA topology. Similar findings were reported upon deletion of *C. fasciculata KAP1* [23] and *A. deanei KAP4*, generating changes in kDNA topology in such double-mutant cells [30]. The data obtained indicate that increased condensation of *A. deanei* kDNA promotes a reduction in cell proliferation. Previous work has demonstrated that various species of trypanosomatids treated with nalidixic acid and other inhibitors of mitochondrial topoisomerase II also exhibited increased kDNA compaction and reduced cell growth [45, 46].

The inability to generate a *KAP7 *null mutant, along with the phenotypic effects observed after single allele deletion, suggests essentiality of this gene in *A. deanei*. By contrast, in epimastigotes of T. cruzi, both *KAP7 *alleles could be deleted without altering kDNA topology [31]. The discrepancies in these findings suggest that KAP7 may play distinct roles and hold varying levels of importance depending on the trypanosomatid species and their respective kDNA arrangements, potentially reflecting differences in mitochondrial physiology and the structural features of kDNA between symbiont-harboring and nonsymbiotic trypanosomatids.

The metabolism of kDNA is regulated by multiple mitochondrial proteins with diverse functions [2, 21, 47]. *KAP7* mutant cells of both species presented alterations in kDNA replication and in *A. deanei*, the appearance of cells with atypical morphologies indicated cytokinesis impairment. Alterations in the kDNA replication process could generate dyskinetoplastic cells. Such phenotype was not observed in this study, probably because the *KAP7* mutants were analyzed in short culture times.

Besides KAPs, more than a hundred proteins participate in kDNA replication. The minicircle replication factor (MiRF172) is crucial for reconnecting molecules to the kDNA network after replication; when depleted in *T. brucei*, cells presented a reduced kDNA content or the dyskinetoplastic phenotype [10]. Cells depleted for mitochondrial Hsp70 or Hsp40 hindered minicircle replication, leading to kDNA loss, thus emphasizing the role of chaperones in kinetoplast structure and function [48].

Interestingly, in *T. cruzi*, the kDNA network of trypomastigotes is less compact than that observed in epimastigotes or amastigotes. Since this life stage is nonreplicative, the relaxed kDNA architecture may reflect reduced demands on replication-associated remodeling. The role of KAPs in trypomastigotes remains unexplored, but it is plausible that their function in these cells diverges from that in replicative stages. Further studies will be required to clarify whether the architectural role of KAP7 extends beyond replication and how it may vary across developmental stages.

Previous data showed that treatment of *A. deanei* with cisplatin did not affect either proliferation or cell cycle progression [30]. However, a decrease in *T. cruzi* cell growth was observed, especially in *KAP7* mutant cells, which did not recover growth after cisplatin treatment. This effect may be associated with the mechanism observed in cancer cells, where cisplatin induces a block in replication and triggers checkpoints in G2/M to allow DNA damage repair. In this case, cell cycle arrest may lead to apoptosis [49–51].

Cisplatin treatment generated symbiont filamentation in *A. deanei KAP7*-mutant cells, as previously reported in protozoa of this species with gene deletion for *KAP4* [30]. Other atypical morphologies were also observed in these protozoa, especially large cells that are usually named as fat cells, as first reported in *T. brucei* [52]. In *A. deanei*, such a phenotype is related to division blockage and symbiont replication impairment [53]. The bacterium filamentation has been associated to alterations in kDNA replication, as observed here, and results in loss of coordinated division between the prokaryote and other DNA-containing structures, such as the kinetoplast and the nucleus [30, 54]. Such division synchrony is a hallmark of symbiosis in trypanosomatids, which represents an excellent model for studying the origin of organelles and cellular evolution [55].

Treatment of *A. deanei* with cisplatin promoted, in both WT and *KAP7* mutant cells, mitochondrial swelling and formation of endoplasmic reticulum profiles, especially around the filamentous symbiont, which may indicate bacterium autophagy. Such phenomena can be associated with the elimination of cell structures with symbiotic origin, such as the mitochondrion (mitophagy) [56]. Similar results were observed in this protozoan after deletion of *KAP4* genes [30]. In *T. cruzi*, prolonged treatment with cisplatin promoted kDNA arrangement that may affect kDNA replication, culminating in the decrease of proliferation observed in mutant cells. According to this idea, in the present work, the morphological analyses of *KAP7* mutants of both species treated with cisplatin revealed enlargement of the posterior region of the cell body, which suggests failures in the cell division process. This is consistent with the effects of this genotoxic agent, which binds to DNA and forms intra- and inter-strand adducts, inhibiting replication and transcription [37].

Exposure to UV induces the formation of DNA photoproducts, causing structural distortions in the double helix that can impair the replication and transcription machinery [57]. Here, the UV exposure affected the proliferation of *KAP7* mutant cells, but not of WT cells, indicating that this protein participates in kDNA metabolism in *A. deanei* and *T. cruzi*. In agreement with this idea, recent data have shown that KAP7 participates in damage response of kDNA lesions caused by UV radiation, and by cisplatin, in both trypanosomatids species [31]. UV did not alter the kinetics of DNA repair in *KAP4* null mutants of *A. deanei*, but it affected the topology of the kinetoplast network upon irradiation [30].

Exposure to UV also induced symbiont filamentation in *A. deanei KAP7* mutants, as previously described to *KAP4* mutants. However, in the latter case, lysed bacteria and aposymbiotic cells were also observed [30]. In the present work, the appearance of mutant cells containing two flagella, one kinetoplast, and two nuclei was consistent with alterations in kDNA replication, as revealed by the TdT technique in *A. deanei* and *T. cruzi*. The higher percentage of protozoa observed in the final phase of kDNA duplication may be associated to kinetoplast division impairment, since, in this stage, the newly replicated minicircles are already connected to the network but remain open [2].

Mammalian cells are generally not capable of repairing UV and cisplatin lesions in mitochondrial DNA [58–60]; however, it is important to mention that several DNA repair proteins previously described as involved in mitochondrial metabolism perform non-canonical functions [61]. Cockayne syndrome A (CSA) and Cockayne syndrome group B (CSB) proteins, for example, are involved in unexpected effects in mitochondrial metabolism [62]. *T. cruzi* exhibits efficient repair mechanisms for kDNA damage caused by various genotoxic agents, including benzonidazole, reactive oxygen species, and methyl methanesulfonate (MMS) [32, 34, 63, 64]. However, the mechanisms involved in kDNA repair in response to lesions caused by UV and cisplatin are not yet fully understood in trypanosomatids [31, 65]. KAP proteins are associated with several distinct kDNA metabolism processes, as transcription and replication [20, 23, 29]. In *T. cruzi*, there is evidence that the CSB, a protein that has a pivotal role in the recovery of RNA synthesis, and KAP7 are involved in transcription-associated kDNA damage response [31]. These data indicate that trypanosomatids have specialized mechanisms for mitochondrial DNA repair that would be related to the evolutionary adaptation capacity of these parasites.

## Conclusions

Different phenotypes for the depletion of the same KAP protein in distinct organisms are already documented in literature. Deletion of *KAP2* and *KAP3* loci in *Crithidia fasciculata* significantly altered cell proliferation [24], but a similar effect was not observed in *KAP3* null mutants of *T. cruzi* [28]. Previous genomic analyzes showed that the *KAP7* sequence is similar in different species, reinforcing the idea that proteins associated with DNA are evolutionarily conserved [29]. In *A. deanei*, KAP7 is an essential protein, and deletion of a single allele led to high kDNA compaction, a phenotype not observed in *T. cruzi KAP7* null mutants. In *T. cruzi*, however, cell proliferation was more severely affected following exposure to genotoxic agents such as cisplatin and UV, corroborating the role of this protein in mitochondrial DNA damage response [31]. These findings suggest that KAP7 may have species-specific functions depending on kDNA organization.

## Supplementary Information

Additional file 1. Figure S1: Gene delete confirmation and proliferation assessment in *A. deanei* and *T. cruzi*mutants. (A) PCR confirmation of gene deletion and resistance marker integration (NEO) in *A. deanei*. (B) Proliferation assessment of *A. deanei* wild-type (WT) and *Δkap7/KAP7* mutant strain. (C) PCR confirmation of gene deletion and resistance markers integration (*HYG and NEO*) in *T. cruzi*. (D) Proliferation assessment of *T. cruzi* wild-type (WT) and *Δkap7*::NEO/*Δkap7*::HYG mutant strains. In panels A and C, agarose gel electrophoresis images display PCR products confirming gene deletion and integration of resistance markers. Lane annotations indicate relevant gene targets and markers. Proliferation data is presented as mean values ± standard deviation (SD) from 3 independent experiments.

Additional file 2. Figure S2: Ultrastructure and morphology of *A. deanei* KAP7 mutants (AdΔkap7/KAP7). Transmission electron microscopy (TEM, panels a–h) and scanning electron microscopy (SEM, panels i–l) images of mutant cells. The square brackets indicate regions of more densely packed kinetoplast DNA (kDNA). The black arrows highlight endoplasmic reticulum profiles surrounding the symbiont (a, c) and ruptured kDNA fibrils (d). The white arrowheads mark cells exhibiting defective cytokinesis. Abbreviations: bb – basal body; f – flagellum; k – kinetoplast DNA; n – nucleus; s – symbiont.

Additional file 3. Table S1: List of oligonucleotides used to confirm null mutants of *KAP7* in *A. deanei* and *T. cruzi*. Sequences are written in the 5’ to 3’ orientation and in bold are highlighted the restriction sites

## Data Availability

Data supporting the main conclusions of this study are included in the manuscript.

## References

[1] Shapiro TA, Englund PT. The structure and replication of kinetoplast DNA. Ann Rev Microbiol. 1995;49:117–43. 10.1146/annurev.mi.49.100195.001001. 8561456 10.1146/annurev.mi.49.100195.001001

[2] Jensen RE, Englund PT. Network news: the replication of kinetoplast DNA. Ann Rev Microbiol. 2012;66:473–91. 10.1146/annurev-micro-092611-150057. 22994497 10.1146/annurev-micro-092611-150057

[3] Melendy T, Sheline C, Ray DS. Localization of a type II DNA topoisomerase to two sites at the periphery of the kinetoplast DNA of *Crithidia fasciculata*. Cell. 1988;55:1083–8. 10.1016/0092-8674(88)90252-8. 2849507 10.1016/0092-8674(88)90252-8

[4] Ryan KA, Englund PT. Synthesis and processing of kinetoplast DNA minicircles in *Trypanosoma equiperdum*. Mol Cell Biol. 1989;9:3212–7. 10.1128/mcb.9.8.3212-3217. 2552285 10.1128/mcb.9.8.3212-3217.1989 PMC362365

[5] Ogbadoyi EO, Robinson DR, Gull K. A high-order trans-membrane structural linkage is responsible for mitochondrial genome positioning and segregation by flagellar basal bodies in trypanosomes. Mol Biol Cell. 2003;14:1769–79. 10.1091/mbc.e02-08-0525. 12802053 10.1091/mbc.E02-08-0525 PMC165075

[6] Schneider A, Ochsenreitter T. Failure is not an option – Mitochondrial genome segregation in trypanosomes. J Cell Sci. 2018;131:jcs221820. 10.1242/jcs.221820. 30224426 10.1242/jcs.221820

[7] Amodeo S, Bregy I, Hoffmann A, Fradera Sola A, Kern M, Baudouin H, et al. Characterization of two novel proteins involved in mitochondrial DNA anchoring in *Trypanosoma brucei*. PLoS Pathog. 2023;19:e1011486. 10.1371/journal.ppat.1011486. 37459364 10.1371/journal.ppat.1011486 PMC10374059

[8] Mensa-Wilmot K, Hoffman B, Wiedman J, Sullenberger C, Sharma A. Kinetoplast division factors in a trypanosome. Trends Parasitol. 2019;35:119–28. 10.1016/j.pt.2018.11.002. 30638954 10.1016/j.pt.2018.11.002 PMC6368890

[9] Guilbride DL, Englund PT. The replication mechanism of kinetoplast DNA networks in several trypanosomatid species. J Cell Sci. 1998;111:675–9. 10.1242/jcs.111.6.675. 9471996 10.1242/jcs.111.6.675

[10] Amodeo S, Jakob M, Ochsenreiter T. Characterization of the novel mitochondrial genome replication factor MiRF172 in *Trypanosoma brucei*. J Cell Sci. 2018;131:1–12. 10.1242/jcs.211730. 10.1242/jcs.211730 PMC5963845 29626111

[11] Carpenter LR, Englund PT. Kinetoplast maxicircle DNA replication in *Crithidia fasciculata* and *Trypanosoma brucei*. Mol Cell Biol. 1995;15:6794–803. 10.1128/MCB.15.12.6794. 8524245 10.1128/mcb.15.12.6794 PMC230933

[12] Li Z, Lindsay ME, Motyka SA, Englund PT, Wang CC. Identification of a bacterial-like HslVU protease in the mitochondria of *Trypanosoma brucei* and its role in mitochondrial DNA replication. PLoS Path. 2008;4:e1000048. 10.1371/journal.ppat.1000048. 10.1371/journal.ppat.1000048 PMC2277460 18421378

[13] Liu B, Wang J, Yaffe N, Lindsay ME, Zhao Z, Zick A, et al. Trypanosomes have six mitochondrial DNA helicases with one controlling kinetoplast maxicircle replication. Mol Cell. 2009;35:490–501. 10.1016/j.molcel.2009.07.004. 19646907 10.1016/j.molcel.2009.07.004 PMC2763077

[14] González A, Rosales JL, Ley V, Díaz C. Cloning and characterization of a gene coding for a protein (KAP) associated with the kinetoplast of epimastigotes and amastigotes of *Trypanosoma cruzi*. Mol Biochem Parasitol. 1990;40:233–43. 10.1016/0166-6851(90)90045-n. 1694571 10.1016/0166-6851(90)90045-n

[15] Cavalcanti DP, Thiry M, De Souza W, Motta MCM. The kinetoplast ultrastructural organization of endosymbiont-bearing trypanosomatids as revealed by deep-etching, cytochemical and immunocytochemical analysis. Histoch Cell Biol. 2008;130:1177–85. 10.1007/s00418-008-0450-7. 10.1007/s00418-008-0450-7 18542983

[16] Gonçalves CS, Ávila AR, De Souza W, Motta MCM, Cavalcanti DP. Revisiting the *Trypanosoma cruzi* metacyclogenesis: morphological and ultrastructural analyses during cell differentiation. Parasit Vectors. 2018;11:83. 10.1186/s13071-018-2664-4. 29409544 10.1186/s13071-018-2664-4 PMC5801705

[17] Zuma AA, Barrias ES, de Souza W. Basic biology of *Trypanosoma cruzi*. Curr Pharm Des. 2021;27:1671–732. 10.2174/1381612826999201203213527. 33272165 10.2174/1381612826999201203213527

[18] Michieletto D. Kinetoplast DNA: a polymer physicist’s topological Olympic dream. Nucleic Acids Res. 2025;53:gkae1206. 10.1093/nar/gkae1206. 39676656 10.1093/nar/gkae1206 PMC11754639

[19] Xu CW, Hines JC, Enge ML, Russel DG, Ray DS. Nucleus-encoded histone H1-like proteins are associated with kinetoplast DNA in the Trypanosomatid *Crithidia fasciculata*. Mol Cel Biol. 1996;16:564–76. 10.1128/MCB.16.2.564. 10.1128/mcb.16.2.564 PMC231035 8552084

[20] Wang J, Pappas-Brown V, Englund PT, Jensen RE. TbKAP6, a mitochondrial HMG box-containing protein in *Trypanosoma brucei*, is the first trypanosomatid kinetoplast-associated protein essential for kinetoplast DNA replication and maintenance. Eukaryot Cell. 2014;13:919–32. 10.1128/EC.00260-13. 24879122 10.1128/EC.00260-13 PMC4135736

[21] Pyrih J, Hammond H, Alves A, Dean S, Sunter JD, Wheeler RJ, et al. Comprehensive sub-mitochondrial protein map of the parasitic protist *Trypanosoma brucei* defines critical features of organellar biology. Cell Rep. 2023;42:113083. 10.1016/j.celrep.2023.11308.3. 37669165 10.1016/j.celrep.2023.113083

[22] Xu C, Ray DS. Isolation of proteins associated with kinetoplast DNA networks *in vivo*. Proc Nat Acad Sci USA. 1993;90:1786–9. 10.1073/pnas.90.5.1786. 8446592 10.1073/pnas.90.5.1786 PMC45964

[23] Lukes J, Hines JC, Evans CJ, Avliyakulov NK, Prabhu VP, Chen J, et al. Disruption of the *Crithidia fasciculata* KAP1 gene results in structural rearrangement of the kinetoplast disc. Mol Biochem Parasitol. 2001;117:179–86. 10.1016/s0166-6851(01)00348-6. 11606228 10.1016/s0166-6851(01)00348-6

[24] Avliyakulov NK, Lukes J, Ray DS. Mitochondrial histone-like DNA-binding proteins are essential for normal cell growth and mitochondrial function in *Crithidia fasciculata*. Euk Cell. 2004;3:518–26. 10.1128/EC.3.2.518-526.2004. 10.1128/EC.3.2.518-526.2004 PMC387644 15075280

[25] Souto-Padron T, De Souza W. Ultrastructural localization of basic proteins in *Trypanosoma cruzi*. J Histochem Cytochem. 1979;26:349–58. 10.1177/26.5.77871. 10.1177/26.5.77871 77871

[26] Zavala-Castro JE, Acosta-Viana K, Guzmán-Marín E, Rosado-Barrera ME, Rosales-Encina JL. Stage specific kinetoplast DNA-binding proteins in *Trypanosoma cruzi*. Acta Trop. 2000;76:139–46. 10.1016/S0001-706X(00)00079-6. 10936573 10.1016/s0001-706x(00)00079-6

[27] Cavalcanti DP, Shimada MK, Probst CM, Souto-Padrón TCBS, De Souza W, Goldenberg S, et al. Expression and subcellular localization of kinetoplast-associated proteins in the different developmental stages of *Trypanosoma cruzi*. BMC Microbiol. 2009;9:120. 10.1186/1471-2180-9-120. 19497120 10.1186/1471-2180-9-120 PMC2700280

[28] de Souza FSP, Rampazzo RDCP, Manhaes L, Soares MJ, Cavalcanti DP, Krieger MA, et al. Knockout of the gene encoding the kinetoplast-associated protein 3 (KAP3) in *Trypanosoma cruzi*: effect on kinetoplast organization, cell proliferation and differentiation. Mol Biochem Parasitol. 2010;172:90–8. 10.1016/j.molbiopara.2010.03.014. 20363262 10.1016/j.molbiopara.2010.03.014

[29] de Souza SS, Catta-Preta CMC, Alves JMP, Cavalcanti DP, Teixeira MMG, Camargo EP, et al. Expanded repertoire of kinetoplast associated proteins and unique mitochondrial DNA arrangement of symbiont-bearing trypanosomatids. PLoS ONE. 2017;12:e0187516. 10.1371/journal.pone.0187516. 29131838 10.1371/journal.pone.0187516 PMC5683618

[30] Gonçalves CS, Catta-Preta CMC, Repolês B, Mottram JC, De Souza W, Machado CR, et al. Importance of *Angomonas deanei* KAP4 for kDNA arrangement, cell division and maintenance of the host-bacterium relationship. Sci Rep. 2021;11:9210. 10.1038/s41598-021-88685-8. 33911164 10.1038/s41598-021-88685-8 PMC8080567

[31] Repolês BM, Ferreira WRR, Assis AV, Mendes IC, Morini FS, Gonçalves CS, et al. Transcription coupled repair occurrence in *Trypanosoma cruzi* mitochondria. Mitochondrion. 2025;22:102009. 10.1016/j.mito.2025.102009. 10.1016/j.mito.2025.102009 39993491

[32] Furtado C, Kunrath-Lima M, Rajão MA, Mendes IC, de Moura MB, Campos PC, et al. Functional characterization of 8-oxoguanine DNA glycosylase of *Trypanosoma cruzi*. PLoS ONE. 2012;7:e42484. 10.1371/journal.pone.0042484. 22876325 10.1371/journal.pone.0042484 PMC3411635

[33] Aguiar PHN, Furtado C, Repolês BM, Ribeiro GA, Mendes IC, Peloso EF, et al. Oxidative stress and DNA lesions: the role of 8-oxoguanine lesions in *Trypanosoma cruzi* cell viability. PLoS Neglect Trop Dis. 2013;7:e2279. 10.1371/journal.pntd.0002279. 10.1371/journal.pntd.0002279 PMC3681716 23785540

[34] Kunrath-Lima M, Repolês BM, Alves CL, Furtado C, Rajão MA, Macedo AM, et al. Characterization of *Trypanosoma cruzi* MutY DNA glycosylase ortholog and its role in oxidative stress response. Infect Gen Evol. 2017;55:332–42. 10.1016/j.meegid.2017.09.030. 10.1016/j.meegid.2017.09.030 28970112

[35] Mitchell DL, Jen J, Cleaver JE. Relative induction of cyclobutane dimers and cytosine photohydrates in DNA irradiated *in vitro* and *in vivo* with ultraviolet-C and ultraviolet-B light. Photochem Photobiol. 1991;54:741–6. 10.1111/j.1751-1097.1991.tb02084.x. 1665910 10.1111/j.1751-1097.1991.tb02084.x

[36] Hu J, Adar S. The cartography of UV-induced DNA damage formation and DNA repair. Photochem Photobiol. 2017;93:199–206. 10.1111/php.12668. 27861959 10.1111/php.12668 PMC5315582

[37] Siddik ZH. Cisplatin: mode of cytotoxic action and molecular basis of resistance. Oncogene. 2003;22:7265–79. 10.1038/sj.onc.1206933. 14576837 10.1038/sj.onc.1206933

[38] Wang D, Lippard SJ. Cellular processing of platinum anticancer drugs. Nat Rev Drug Disc. 2005;4:307–20. 10.1038/nrd1691. 10.1038/nrd1691 15789122

[39] Warren LG. Metabolism of *Schizotrypanum cruzi* Chagas. I. Effect of culture age and substrate concentration on respiratory rate. J Parasitol. 1960;46:529–39. 10.2307/3274932. 13783227

[40] Camargo EP. Growth and differentiation in *Trypanosoma cruzi.* I. Origin of metacyclic trypanosomes in liquid media. Rev Inst Med Trop São Paulo. 1964;6:93–100. 14177814

[41] Andrade IS, Vianez-Júnior JL, Goulart CL, Homblé F, Ruysschaer J, von Krüge WMA, et al. Characterization of a porin channel in the endosymbiont of the trypanosomatid protozoan *Crithidia deanei*. Microbiol. 2011;157:2818–30. 10.1099/mic.0.049247-0. 10.1099/mic.0.049247-0 21757490

[42] Liu B, Englund PT. The rotational dynamics of kinetoplast DNA replication. Mol Microbiol. 2007;64:676–90. 10.1111/j.1365-2958.2007.05686.x. 17462016 10.1111/j.1365-2958.2007.05686.x

[43] Amodeo S, Bregy I, Ochsenreiter T. Mitochondrial genome maintenance – The kinetoplast story. FEMS Microbiol Rev. 2023;47:1–12. 10.1093/femsre/fuac047. 10.1093/femsre/fuac047 PMC10719067 36449697

[44] Cadena R, Svobodová M, Benz C, Rašková V, Chmelová L, Škodová-Sveráková I, et al. Characterization of novel and essential kinetoplast-associated proteins in *Trypanosoma brucei*. Biorxiv. 2023. 10.1101/2024.04.22.590512.36993218

[45] Cavalcanti DP, Fragoso SP, Goldenberg S, De Souza W, Motta MCM. The effect of topoisomerase II inhibitors on the kinetoplast ultrastructure. Parasitol Res. 2004;94:439–48. 10.1007/s00436-004-1223-4. 15517387 10.1007/s00436-004-1223-4

[46] Balaña-Fouce RAV, Fernández-Prada C, García-Estrada C, Reguera RM. Trypanosomatids topoisomerase re-visited. New structural findings and role in drug discovery. Int J Parasitol Drugs Drug Resist. 2014;4:326–37. 10.1016/j.ijpddr.2014.07.006. 25516844 10.1016/j.ijpddr.2014.07.006 PMC4266802

[47] Povelones ML, Beyond replication,. Division and segregation of mitochondrial DNA in kinetoplastids. Mol Biochem Parasitol. 2014;196:53–60. 10.1016/j.molbiopara.2014.03.008. 24704441 10.1016/j.molbiopara.2014.03.008

[48] Týč J, Klingbeil MM, Lukeš J. Mitochondrial heat shock protein machinery Hsp70/Hsp40 is indispensable for proper mitochondrial DNA maintenance and replication. MBio. 2015;6:1–14. 10.1128/mbio.02425-14. 10.1128/mBio.02425-14 PMC4337576 25670781

[49] Sorenson CM, Barry MA, Eastman A. Analysis of events associated with cell cycle arrest at G_2_ phase and cell death induced by cisplatin. J Nat Cancer Inst. 1990;82:749–55. 10.1093/jnci/82.9.749. 1691303 10.1093/jnci/82.9.749

[50] Qin LF, Ng IOL. Induction of apoptosis by cisplatin and its effect on cell cycle-related proteins and cell cycle changes in hepatoma cells. Cancer Let. 2002;175:27–38. 10.1016/S0304-3835(01)00720-0. 11734333 10.1016/s0304-3835(01)00720-0

[51] Sarin N, Engel F, Kalayda GV, Mannewitz M, Cinatl J Jr, Rothweiler F, et al. Cisplatin resistance in non-small cell lung cancer cells is associated with an abrogation of cisplatin-induced G_2_/M cell cycle arrest. PLoS ONE. 2017;12:e0181081. 10.1371/journal.pone.0181081. 28746345 10.1371/journal.pone.0181081 PMC5528889

[52] Shi H, Djikeng A, Mark T, Wirtz E, Tschudi C, Ullu E. Genetic interference in *Trypanosoma brucei* by heritable and inducible double-stranded RNA. RNA. 2000;6:1069–76. 10.1017/s1355838200000297. 10917601 10.1017/s1355838200000297 PMC1369981

[53] Catta-Preta CMC, Pascoalino BS, de Souza W, Mottram JC, Motta MCM, Schenkman S. Reduction of tubulin expression in *Angomonas deanei* by RNAi modifies the ultrastructure of the trypanosomatid protozoan and impairs division of its endosymbiotic bacterium. J Euk Microbiol. 2016;63:794–803. 10.1111/jeu.12326. 27194398 10.1111/jeu.12326

[54] Catta-Preta CMC, Brum FL, da Silva CC, Zuma AA, Elias MC, de Souza W, et al. Endosymbiosis in trypanosomatid protozoa: the bacterium division is controlled during the host cell cycle. Front Microbiol. 2015;6:520. 10.3389/fmicb.2015.00520. 26082757 10.3389/fmicb.2015.00520 PMC4451579

[55] Motta MCM, Catta-Preta CMC, Schenkman S, Martins ACDA, Miranda K, de Souza W, et al. The bacterium endosymbiont of *Crithidia deanei* undergoes coordinated division with the host cell nucleus. PLoS ONE. 2010;5:e12415. 10.1371/journal.pone.0012415. 20865129 10.1371/journal.pone.0012415 PMC2932560

[56] Palikaras K, Lionaki E, Tavernarakis N. Mechanisms of mitophagy in cellular homeostasis, physiology and pathology. Nat Cell Biol. 2018;20:1013–22. 10.1038/s41556-018-0176-2. 30154567 10.1038/s41556-018-0176-2

[57] Rastogi RP, Richa KA, Tyagi MB, Sinha RP. Molecular mechanisms of ultraviolet radiation-induced DNA damage and repair. J Nucl Acids. 2010;16:592980. 10.4061/2010/592980. 10.4061/2010/592980 PMC3010660 21209706

[58] Clayton DA, Doda JN, Friedberg EC. The absence of a pyrimidine dimer repair mechanism in mammalian mitochondria. Proc Nat Acad Sci USA. 1974;71:2777–81. 10.1073/pnas.71.7.2777. 4212385 10.1073/pnas.71.7.2777 PMC388554

[59] Pascucci B, Versteegh A, van Hoffen A, van Zeeland AA, Mullenders LHF, Dogliotti E. DNA repair of UV photoproducts and mutagenesis in human mitochondrial DNA. J Mol Biol. 1997;273:417–27. 10.1006/jmbi.1997.1268. 9344749 10.1006/jmbi.1997.1268

[60] Podratz JL, Knight AM, Ta LE, Staff NP, Gass JM, Genelin K, et al. Cisplatin induced mitochondrial DNA damage in dorsal root ganglion neurons. Neurobiol Dis. 2011;41:661–8. 10.1016/j.nbd.2010.11.017. 21145397 10.1016/j.nbd.2010.11.017 PMC3031677

[61] Scheibye-Knudsen M, Ramamoorthy M, Sykora P, Maynard S, Minor LP, RK, et al. Cockayne syndrome group B protein prevents the accumulation of damaged mitochondria by promoting mitochondrial autophagy. J Exp Med. 2012;209:855–69. 10.1084/jem.20111721. 22473955 10.1084/jem.20111721 PMC3328359

[62] Kamenisch Y, Fousteri M, Knoch J, von Thaler A, Fehrenbacher B, Kato H, et al. Proteins of nucleotide and base excision repair pathways interact in mitochondria to protect from loss of subcutaneous fat, a hallmark of aging. J Exp Med. 2010;207:379–90. 10.1084/jem.20091834. 20100872 10.1084/jem.20091834 PMC2822596

[63] Rajão MA, Furtado C, Alves CL, Passos-Silva DG, De Moura MB, Schamber-Reis BL, et al. Unveiling Benznidazole’s mechanism of action through overexpression of DNA repair proteins in *Trypanosoma cruzi*. Environ Mol Mutagen. 2014;55:309–21. 10.1002/em.21839. 24347026 10.1002/em.21839

[64] Martins TMAS, Peloso EF, Costa-Silva HM, Rajão MA, Van Houten B, Machado CR. Mitochondrial behavior during nuclear and mitochondrial DNA repair in *Trypanosoma cruzi* epimastigotes. Exp Parasitol. 2020;219:108016. 10.1016/j.exppara.2020.108016. 33035543 10.1016/j.exppara.2020.108016

[65] Machado-Silva A, Cerqueira PG, Grazielle-Silva V, Gadelha FR, Peloso EF, Teixeira SMR, et al. How Trypanosoma cruzi deals with oxidative stress: antioxidant defence and DNA repair pathways. Mutat Res Rev Mutat Res. 2016;767:8–22. 10.1016/j.mrrev.2015.12.003. 27036062 10.1016/j.mrrev.2015.12.003

[66] Contreras VT, Araujo-Jorge TC, Bonaldo MC, Thomaz N, Barbosa HS, Meirelles M de N, Goldenberg S. Biological aspects of the Dm 28c clone of Trypanosoma cruzi after metacyclogenesis in chemically defined media. Mem Inst Oswaldo Cruz, 1988; 83:123–33. 10.1590/S0074-02761988000100016. 10.1590/s0074-02761988000100016 3074237

[67] Contreras VT, Salles JM, Thomas N, Morel CM, Goldenberg S. In vitro differentiation of Trypanosoma cruzi under chemically defined conditions. Mol Biochem Parasitol, 1985;16:315–327. 10.1016/0166-6851(8590073-8. 10.1016/0166-6851(85)90073-8 3903496

