# Supplementary figures and images for: From mitochondrial DNA arrangement to repair: a kinetoplast-associated protein with different roles in two trypanosomatid species
Source: Parasit Vectors. 2025 Aug 28;18:366. doi: 10.1186/s13071-025-06985-8 (PMC12395728; doi:10.1186/s13071-025-06985-8)

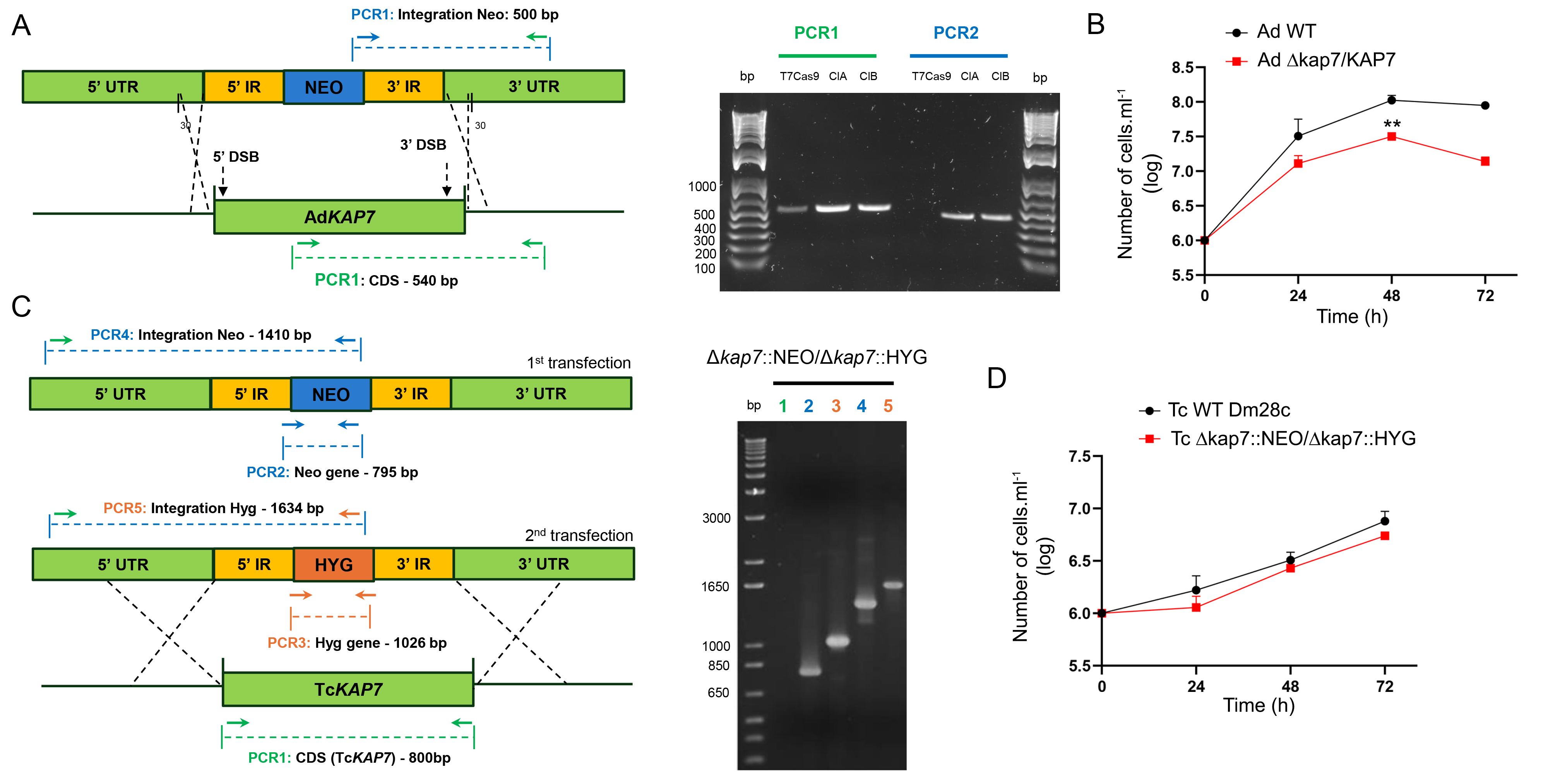

Supplement: Supplementary file 1 — Additional file 1. Figure S1: Gene delete confirmation and proliferation assessment in A. deanei and T. cruzimutants. (A) PCR confirmation of gene deletion and resistance marker integration (NEO) in A. deanei. (B) Proliferation assessment of A. deanei wild-type (WT) and Δkap7/KAP7 mutant strain. (C) PCR confirmation of gene deletion and resistance markers integration (HYG and NEO) in T. cruzi. (D) Proliferation assessment of T. cruzi wild-type (WT) and Δkap7::NEO/Δkap7::HYG mutant strains. In panels A and C, agarose gel electrophoresis images display PCR products confirming gene deletion and integration of resistance markers. Lane annotations indicate relevant gene targets and markers. Proliferation data is presented as mean values ± standard deviation (SD) from 3 independent experiments. [file 13071_2025_6985_MOESM1_ESM.jpeg]

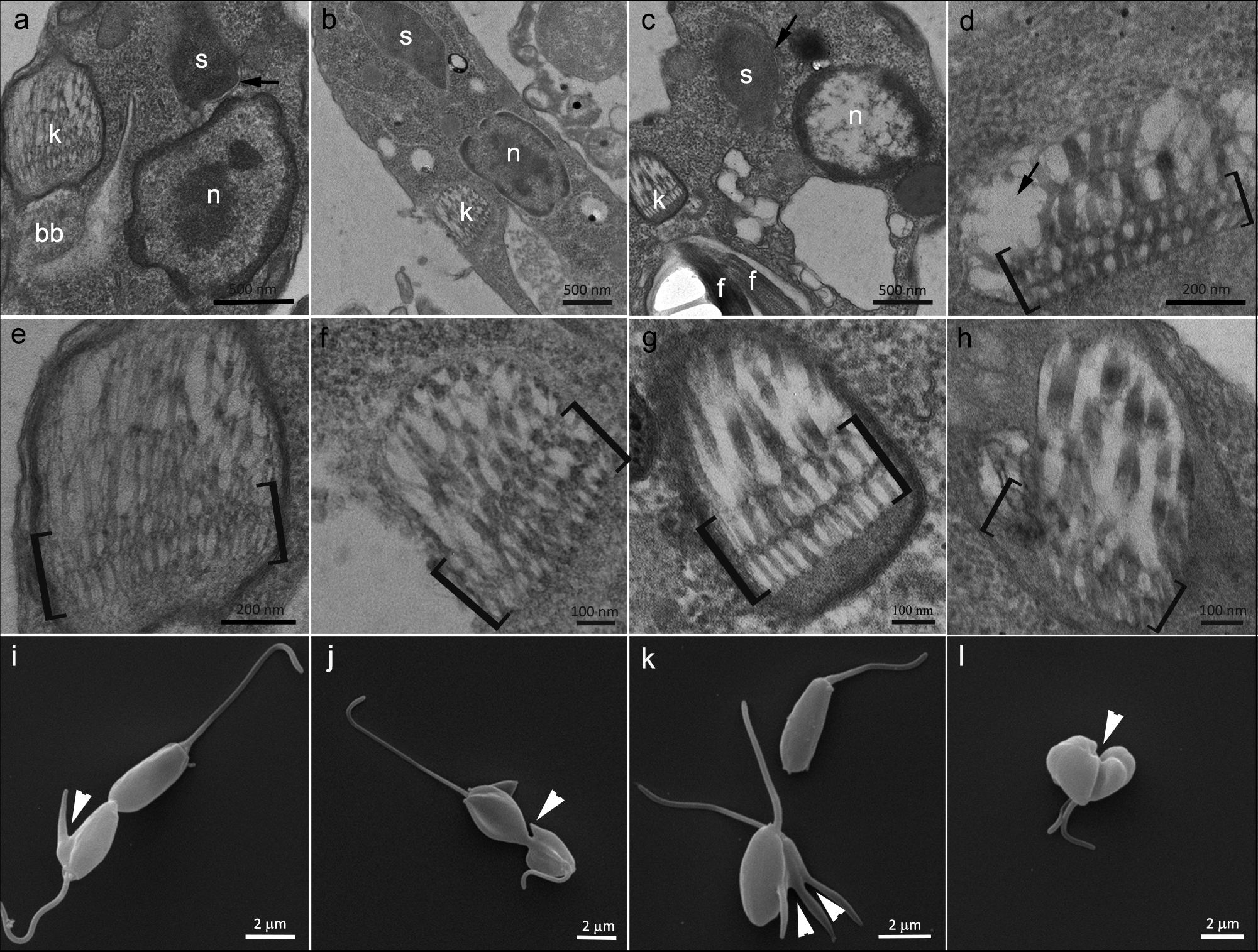

Supplement: Supplementary file 2 — Additional file 2. Figure S2: Ultrastructure and morphology of A. deanei KAP7 mutants (AdΔkap7/KAP7). Transmission electron microscopy (TEM, panels a–h) and scanning electron microscopy (SEM, panels i–l) images of mutant cells. The square brackets indicate regions of more densely packed kinetoplast DNA (kDNA). The black arrows highlight endoplasmic reticulum profiles surrounding the symbiont (a, c) and ruptured kDNA fibrils (d). The white arrowheads mark cells exhibiting defective cytokinesis. Abbreviations: bb – basal body; f – flagellum; k – kinetoplast DNA; n – nucleus; s – symbiont. [file 13071_2025_6985_MOESM2_ESM.jpg]
