# Supplementary material for: From mitochondrial DNA arrangement to repair: a kinetoplast-associated protein with different roles in two trypanosomatid species
Source: Parasit Vectors. 2025 Aug 28;18:366. doi: 10.1186/s13071-025-06985-8 (PMC12395728; doi:10.1186/s13071-025-06985-8)
Supplement: Supplementary file 3 — Additional file 3. Table S1: List of oligonucleotides used to confirm null mutants of KAP7 in A. deanei and T. cruzi. Sequences are written in the 5’ to 3’ orientation and in bold are highlighted the restriction sites [file 13071_2025_6985_MOESM3_ESM.docx]

| **Oligo Name** | **Description** | **Sequence** |
| --- | --- | --- |
| **OL00** | Universal reverse primer for sgRNA amplification - Reverse | aaaagcaccgactcggtgccactttttcaagttgataacggactagccttattttaacttgctatttctagctctaaaac |
| **OL08** | AdKAP7 - 5' sgRNA primer - Forward | gaaattaatacgactcactataggACTTCTCGCTTCTGCCTCGAgttttagagctagaaatagc |
| **OL09** | AdKAP7 - 3' sgRNA primer - Forward | gaaattaatacgactcactataggCCGCCGTCGTTCCTCACTGAgttttagagctagaaatagc |
| **OL10** | AdKAP7 - Upstream Forward primer | TATGTTCCGTACTTCTCGCTTCTGCCTCGAgtataatgcagacctgctgc |
| **OL11** | AdKAP7 - Downstream Reverse primer | TTTCACTCGGCACCTCCGCCGTCGTTCCTCccaatttgagagacctgtgc |
| **OL12** | AdKAP7 – Checking CDS presence/deletion/ Neo integration - Forward | CGGTGCTGCCGCCGGTTT |
| **OL13** | AdKAP7 – Checking CDS presence/deletion - Reverse | CTTCATCTTCTCCGGCTGCG |
| **OL14** | Neomycin resistance gene 3’end - Forward | GCCTTCTATCGCCTTCTTGAC |
| **UPSKAP7F** | TcKAP7 - Upstream region cloning with Neomycin gene vector with SalI restriction site - Forward | GGG**GTCGAC**CGTTGCTGGTTTTTCTTTTGGTGT |
| **UPSKAP7R** | TcKAP7 - Upstream region cloning with Neomycin gene vector with HindIII restriction site - Reverse | GGG**AAGCTT**GCTTCAAAACGTCTATGCGGGC |
| **DOWNKAP7F** | TcKAP7 - Downstream region cloning with Neomycin gene vector with EcoRI restriction site - Forward | GGG**GAATTC**GCCTCATATGACGCATCTCCCA |
| **DOWNKAP7R** | TcKAP7 - Downstream region cloning with Neomycin gene vector with BamHI restriction site - Reverse | GGG**GGATCC**TGTTGGCGCTGTCAAGGAAGTAAG |
| **HygroF** | Cloning of Hygromycin with HindIII site in TcKAP7-Neomycin vector – Forward | GGGGG**AAGCTT**ATGAAAAAGCCTGAACTCACCGCGAC |
| **HygroR** | Cloning of Hygromycin with HindIII site in TcKAP7-Neomycin vector – Reverse | GGG**AAGCTT**CTATTCCTTTGCCCTCGGACGAGTGCTG |
| **EXTF** | Primer upstream integration of KO templates in TcKAP7 endogenous locus -Forward | CCCGCTACGACTGCCCTCCACGCGGAAGGGAA |
| NEOF | Amplification of Neomycin gene - Forward | GGGGAAGCTTATGATTGAACAAGATGGATTGCACGCAG |
| NEOR | Amplification of Neomycin gene - Reverse | GGGGGAATTCAGAAGAACTCGTCAAGAAGGCGATAGAA |
